# Preparation and Characterization of Chitosan Nanofiber: Kinetic Studies and Enhancement of Insulin Delivery System

**DOI:** 10.3390/nano14110952

**Published:** 2024-05-29

**Authors:** Sarah A. Fouad, Amel M. Ismail, M. Abdel Rafea, M. A. Abu Saied, Ali El-Dissouky

**Affiliations:** 1 Chemistry Department, Faculty of Science, Alexandria University, Ibrahimia, Alexandria 21321, Egypt; sarahfouad43@gmail.com (S.A.F.); alieldissouky@alexu.edu.eg (A.E.-D.); 2 Department of Physics, College of Science, Imam Mohammad Ibn Saud Islamic University (IMSIU), Riyadh 11623, Saudi Arabia; makonsow@imamu.edu.sa; 3 Polymeric Materials Research Department, Advanced Technology and New Materials Research Institute, City of Scientific Research and Technological Applications (SRTA-CITY), New Borg El-Arab, Alexandria 21934, Egypt

**Keywords:** chitosan, nanofiber, kinetic, insulin delivery system

## Abstract

Insulin-loaded nanofibers were prepared using chitosan as a natural polymer. The loaded insulin with polyethylene oxide was used for preparing monolayer batch S1. Nanofiber S1 was coated by seven layers of film on both sides to form batch S2 as a sandwich containing Layer A (CS, PEG and PEO) and Layer B (PEG and PEO) using electrospinning apparatus. SEM, TEM and FT-IR techniques were used to confirm the drug loading within the composite nanofibers. The in vitro activity that provided a sustained and controlled release of the drug from the nanofiber batch was studied at different pH values spectrophotometrically using a dialysis method. In batches S1 and S2, the release of insulin from nanofiber proceeds via burst release necessary to produce the desired therapeutic activity, followed by slow step. The rate and the percentage release of insulin in batch S2 are found to be higher at all pH values.

## 1. Introduction

*Diabetes mellitus* is a destructive chronic disease [1,2]. Around the globe, the number of diabetes cases is expected to reach 700 million by 2045 [3]. Although diabetes has been under extensive research for decades, insulin is still the most reliable treatment for diabetes. It is a peptide hormone produced by the pancreas in non-diabetic people and mainly administered to diabetic patients subcutaneously [4,5]. Because of injection-related side effects, exploring other insulin administration pathways is a necessity [6]. Oral delivery would be convenient and less problematic for the patients. Unfortunately, a major obstacle against insulin oral admiration is its liability to degenerate under the effect of the gastrointestinal digestive tract. Therefore, drug carriers that carry insulin that protect and deliver it to the spot of action sound like a very reasonable solution [7,8].

Chitosan (CS) is a nontoxic polysaccharide that is biocompatible, antibacterial, and biodegradable. It binds well to the epithelial tissues and has excellent permeation behavior. Chitosan is generally considered nontoxic and biodegradable, with an oral LD50 in mice of over 16 g/kg. In addition, chitosan has many advantages including safety, biodegradability, ease of modification, ease of DNA or protein complex formation, widespread availability, and low cost, which justify the continuing development of this promising drug and gene delivery system. The safety of chitosan, its ability to prolong residence time in the gastrointestinal tract through mucoadhesion, and its ability to enhance absorption by increasing cellular permeability have all been major factors contributing to its widespread evaluation as a component of oral dosage forms [9,10]. Therefore, CS has been widely applied biomedically and recently drew attention for use as an insulin carrier [11,12]. The positive charges of CS combined with the negative charges of insulin induce formation of an insoluble complex. Nevertheless, CS readily dissolves in acidic media (due to its basic nature, pKa ≈ 6.5) and hence the Cs-insulin insoluble complex disintegrates within the gastric acidity. Polymers have been mixed with CS to render it resistant to the gastric acidity [12,13].

More specifically, with the development of nanotechnologies and nanomaterials, new drug delivery systems (DDS) are being developed. For example, electrohydrodynamic jet printing technology, electro spraying technology, and electrospinning technology are being developed. Among these, electrospinning technology is the most popular technology [14,15,16]. Electrospinning, as a versatile, simple, and cost-effective method to engineer a variety of micro or nanofibrous materials, has contributed to significant developments in the biomedical field [17]. Polymer nanofibers are often used as carriers and widely used in drug delivery due to their high biocompatibility and stability, high specific surface area and volume ratio, high porosity, and high similarity to extracellular matrix (ECM) [18,19]. Currently, the combination of nanotechnology and pharmaceutics has made a possibility the realization of the oral administration of macromolecular drugs, and it is also a promising research direction for oral insulin in order to improve the adaptability of patients and reduce the pain caused by injection, as well as enable the development of noninvasive insulin administration [20,21,22]. 

The first challenge for nanofibers is ensuring stability over a wide range of pH variations in the GI tract. Nanofibers reach the stomach from the mouth in less than 1 min, where they encounter a sudden reduction in pH from 6.8 to 1.2, followed by a transition from a highly acidic environment to a slightly basic environment (pH 6.5 to 8.0) in the small intestine. Studies have shown that the dissociation of the nanoparticulate structure could happen due to the deprotonation or protonation of particle components (such as polysaccharides, proteins, or lipids) in different pH environments, resulting in the burst release and degradation of encapsulated bioactive compounds. Hence, good GI-stability of nanoparticles across a wide range of pH values is crucial to protect insulin throughout the entire GI transition against chemical degradation and improve its oral bioavailability [23].

It is known that chitosan is highly soluble under the acidic environment in the stomach due to the protonation of amine groups. As a result, severe swelling or dissociation of chitosan-based nanofibers could occur, leading to the burst release of insulin. In order to overcome this drawback, this work aims to (i) use nanometric carrier materials as insulin carriers, as well as prepare and characterize electerospun chitosan fibers blended with other polymers for oral insulin delivery and construct multilayers nanofibers consisting of seven layers; (ii) investigate the kinetics of the drug release of insulin in vitro from the nanofibers in an aqueous pH solution, simulating the biological body fluids at the physiological temperature of the body (37 ± 0.5 °C) using UV-visible spectroscopy; and (iii) fit the release data to some common kinetic models. 

## 2. Materials and Method

Insulin “rapid insulin” (In, Egyptian Drug Trading Company, Cairo, Egypt), poly ethylene oxide (PEO, M.W.800,000, Sigma Aldrich, Taufkirchen, Germany), polyethylene glycol (PEG, M.W 1000, Merk, Darmstadt, Germany), chitosan (CS, Highmolecularweight, biobasic, Acros, Janssen-Pharmaceuticalaan, Belgium), Triflorocaceticacid (TFA, Carlo Erba, SABADELL, Alicante, Spain), and heparin calcium (Nile Company, Cairo, Egypt) were used in this study.

## 3. Electrospun Fiber Production

### 3.1. Preparation CS/PEO/In (Batch S1) Solution

Chitosan (16 mg/mL) and PEO (8 mg/mL) were added to TFA (15 mL) as the main layer “S1”. The mixture was stirred for 24 h at room temperature, allowing all solid components to completely dissolve. Then, insulin (10 mg/mL) was added, which was dissolved rapidly to produce the required solution. The solution was electrospun by utilizing the electrospinning apparatus to give solid fiber mats under the following conditions: the voltage was 18 kV, the distance between the drum and the needle was 17 cm, and the flow rate was 0.6 mL/h. The blank of this layer was made without insulin. Fibers were dried under a vacuum to remove residual solvent. 

### 3.2. Preparation of Multilayers Nanofiber (Batch S2) Containing Insulin as a Sandwich

**Figure 1:**
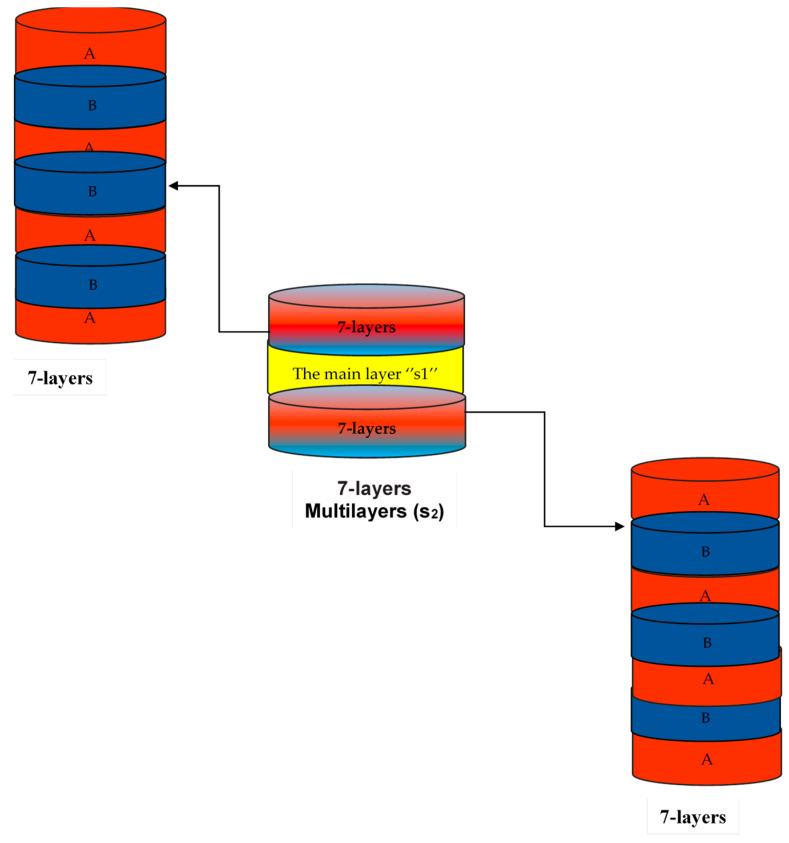
Multilayer nanofibers look like a sandwich: Layer A (CS, PEO, and PEG in TFA) and Layer B (PEO and PEG in heparin), as well as S1, the main layer (CS, PEO, In).

Multilayer nanofibers consisting of seven layers above and below the monolayer S1 in the form of a sandwich, as shown in Figure 1, were constructed from Layer A made from CS, PEG, and PEO dissolved in TFA with the concentrations shown in Table 1. The solution was electrospun into solid fiber mats under the following conditions: the voltage was 20 kV, the distance between the drum and the needle was, 17 cm and there was a 0.65 mL/h flow rate. Layer B made from PEG and PEO dissolved in heparin with the concentrations given in Table 1. The solution was electrospun into solid fiber mats under the following conditions: the voltage was 18 kV, the distance between the drum and the needle was 17 cm, and 0.5 mL/h was the flow rate. The first seven layers were made from A and B reciprocally and then electrospun with fibers from the main layer (S1), and finally, they were made into seven layers again (from A and B reciprocally, as shown below). Mechanical properties and swelling behavior were studied for the prepared nanofibers. 

### 3.3. In Vitro Kinetic Studies of Insulin Release from the Nanofibers

The determination of insulin release profiles from the one layer (S1) and multilayer (S2) prepared from nanofibers were studied spectrophotometrically using a dialysis method. The dialysis bags (VISKING dialysis tubing regenerated cellulose, diameter 21) were soaked before use in distilled water at room temperature for 10 min to remove the preservative, followed by a thorough rinsing in distilled water. We dissolved 20 mg of nanofiber in 1.5 mL of the desired buffer (pH 1.2, pH 6.8, or pH 7.4); then, the dialysis system with the dissolved nanofibers was suspended in 25 mL of the same buffer solution. The suspension was shaken using a water bath shaker at 37 ± 0.5 °C under 100 rpm. A weight of insulin release from nanofibers was studied by withdrawing aliquots of the releasing medium at different scheduled time intervals at λ_max_ 280 nm with a UV-visible spectrophotometer (Jasco, psc-498T, Crestline, OH, USA). The same volume of buffer at the same temperature was added to maintain a constant release volume (25 mL). The length of the dialysis tubing was constant to ensure that the surface area available for dialysis remained unchanged to ensure that a (25:1.5) dilution between the donor and acceptor compartments provided sink conditions. 

## 4. Result and Discussion

### 4.1. Nanofiber Characterization

#### 4.1.1. FTIR Spectra

**Figure 2:**
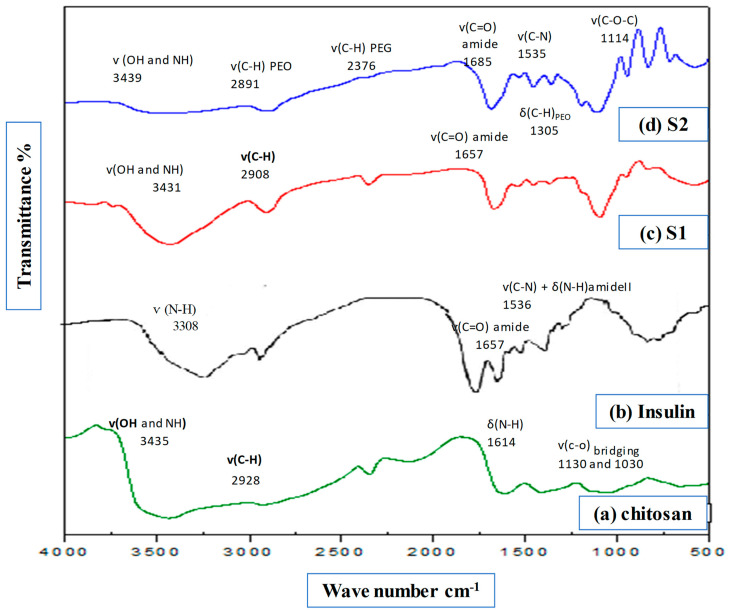
FTIR for (**a**) chitosan, (**b**) insulin, (**c**) S1 (batch 1) and (**d**) S2 (batch 2).

The FTIR spectra of the prepared species are shown in Figure 2. The FTIR spectrum of chitosan, Figure 2a, displays bands at 3435, 2928, 1614, 1130, and 1030 cm^−1^ assigned to v(OH and NH), v(C-H). δ(N-H bend), and v(c-o) bridging, respectively [24,25]. The spectrum shown in Figure 2b exhibits a series of bands characteristic of insulin. These bands are at 3308, 1657, and 1536 cm^−1^ due to v(N-H), v(C=O)_amide_, and v(C-N) + δ(N-H) amide II [26,27]. The infrared spectrum of S1 nanofiber consists of chitosan, insulin and PEO; Figure 2c shows a characteristic band for v(OH and NH) at 3431 cm^−1^, v(C-H)_PEO_ at 2908 cm^−1^, and v(C=O)_amide_ at 1674 cm^−1^.The observed slight shifts in these bands relative to the insulin and chitosan could be referred to as the electrostatic interaction between the negative charges of insulin and positive charges of chitosan [28]. In addition, another series of IR bands is shown at 1541, 1458, and 1305 cm^−1^ due to the amide-II, insulin, and δ(C-H)_PEO_, respectively. The IR band characteristic of v(C-O-C) ether is observed at 1097 cm^−1^ compared with 1130 cm^−1^ in chitosan. With respect to S2, which consists of multilayer nanofiber consisting of seven layers above and below monolayer S1 as a sandwich, seven layers were made from A and B reciprocally and then electrospun with fibers from the main layer (S1) above, before, finally, being made into seven layers again (from A and B reciprocally, as shown below). For Layer A (CS, PEO, and PEG) and Layer B (PEO and PEG), the FTIR spectrum shown in Figure 2d is similar to that of S1, especially the bands characteristic of PEG and PEO. The spectrum displays bands characteristic of v(OH and NH), v(C-H)_PEO_, v(C-H) _PEG_, v(C=O)_amide_, and amide –II in insulin at 3439, 2891, 2376, 1685, and 1535 cm^−1^, respectively. The bands at 1458 cm^−1^ 1357 cm^−1^ are assigned to the v(C-H bending) of PEO and PEG, respectively. The v(C-O-C) band appeared at 1114 cm^−1^.

#### 4.1.2. Scanning Electron Microscopy (SEM)

**Figure 3:**
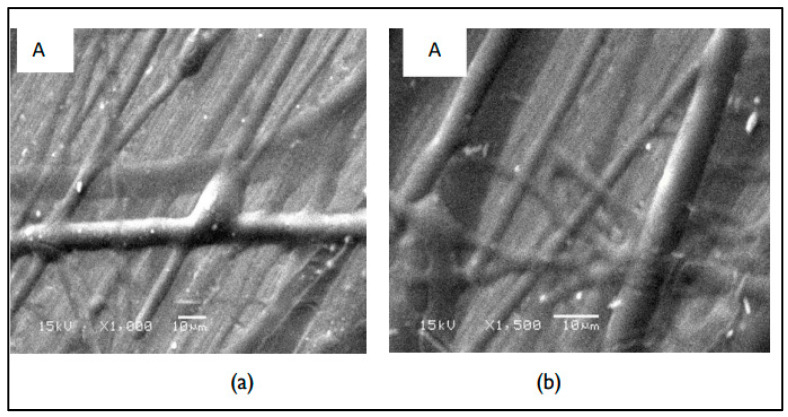
The morphological features of the first-layer electrospun fiber A: (**a**) 1000× and (**b**) 1500×.

**Figure 4:**
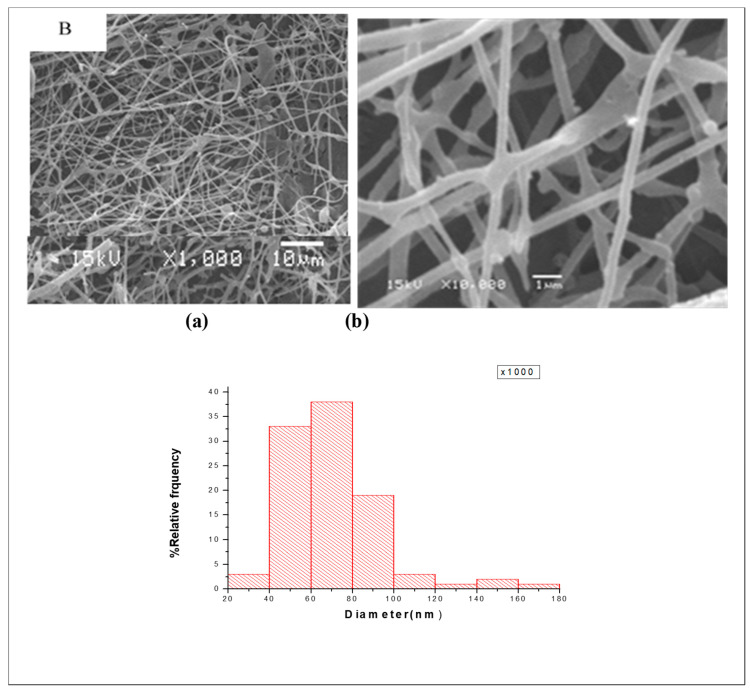
The morphological features and size distribution curve of the second-layer electrospun fiber B: (**a**) 1000× and (**b**) 10,000×.

**Figure 5:**
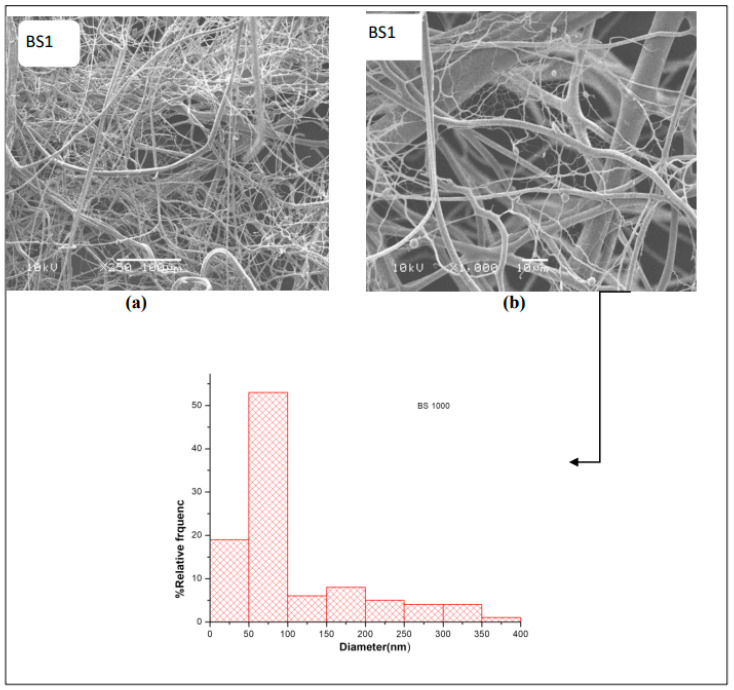
The morphological features and size distribution curve of BS (the blank of one-layer “S1”): (**a**) 250× and (**b**) 1000×.

**Figure 6:**
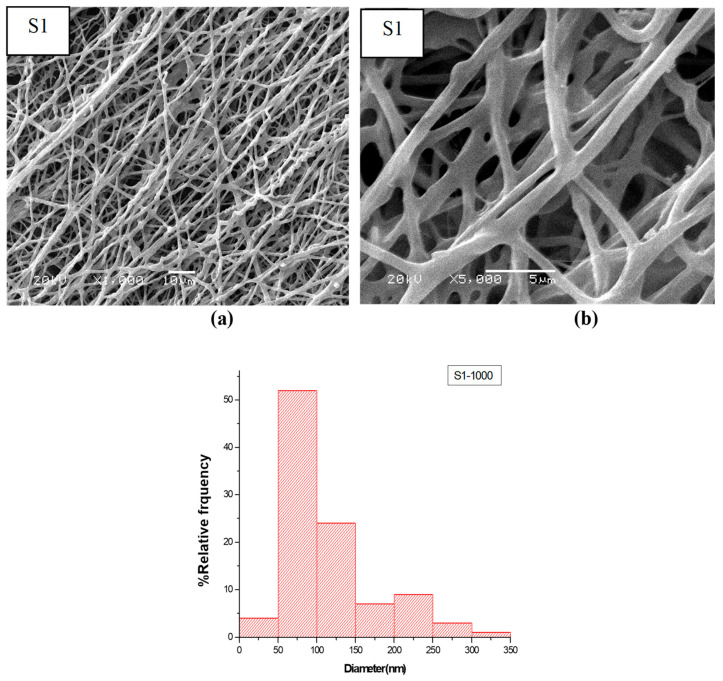
The morphological features and size distribution of electrospun fibers (one-layer “batch S1”): (**a**) 1000× and (**b**) 5000×.

**Figure 7:**
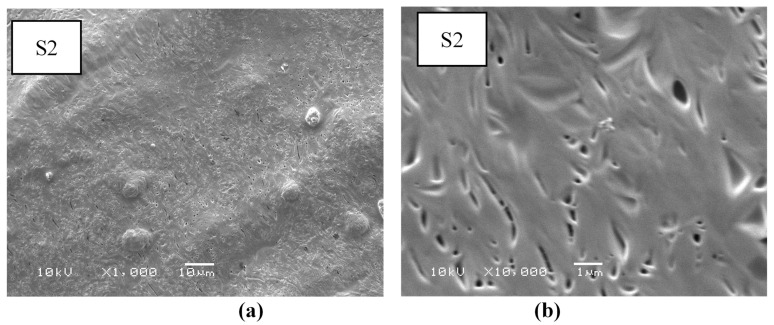
Surface morphology of electrospun layers of fibers (S2): (**a**) 1000× and (**b**) 10,000×.

The morphological features of electrospun fibers are recorded via SEM. The micrographs of the first layer (A) that consists of CS, PEO, and PEG are shown in Figure 3. The micrographs indicate that the surface of this layer consists of thick fibers and is almost smooth with no pores. The micrograph and size distribution showed that the electrospinning of the mixture of PEO and PEG, which are the components of Layer B shown in Figure 4, had a nanofiber structure. The size distribution curve shows that 33% of the fibers have a diameter in the range of 40–60 nm, while 38% of the fibers have a diameter in range of 60–80 nm. The nanofiber structures and the size distribution curve of the blank part of the main layer BS1 (consist of CS and PEO) are shown in Figure 5. The size distribution curve shows that 18% of the diameter is in the range of 0–50 nm and 53% of the diameter is in the range of 50–100 nm. The next SEM image is of the main layer (batchS1), which consists of insulin, CS, and PEO. CS is a supporting material, to which PEO was added to improve the fiber properties [29,30], as shown in the image in Figure 6. The micrographs show nanofibers, and their size distribution curves show that 4% have diameters in the range of 0–50 nm and 52% have diameters in the range of 50–100 nm. The surface morphology of the multilayer (batch S2) consists of seven layers that are made from A and B reciprocally and then electrospun with fibers from the main layer (S1) above, before, finally, being made into seven layers again (from A and B reciprocally below), as shown in Figure 7. The images of the SEM indicated the formation of a mat fiber; the mat fiber structure is very suitable for insulin delivery.

#### 4.1.3. Transmission Electron Microscopy (TEM)

**Figure 8:**
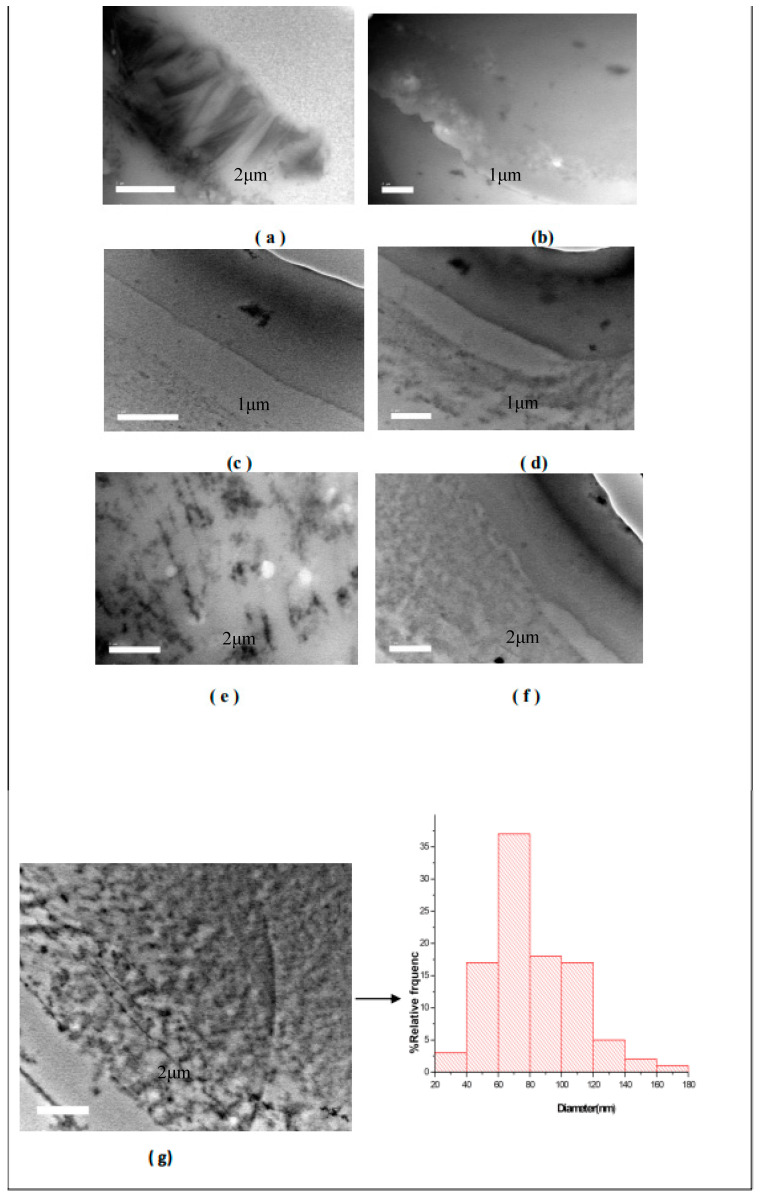
The TEM cross-sectional (**a**–**d**) and surface (**e**–**g**) morphology of electrospun multilayer S2 (batch 2).

The different TEM micrographs for S2 are shown in Figure 8a and display the following pictures. A illustrates the fiber structure of the sample, and Figure 8b–g illustrate the existence of insulin as particles inside the sample S1. The micrograph g shows that 17% of the particles have dimensions in the 20–60 nm range, but 37% have diameters in the range of 60–80 nm. Based on the SEM and TEM data, it was concluded that it is possible to prepare insulin nanoparticles based on CS nanofibers. In addition, the mechanical properties and swelling behavior results are shown in Table 2.

### 4.2. In Vitro Release Study

Insulin release from nanofibers was tested in vitro spectrophotometrically using the dialysis membrane method [31]. Herein, 20 mg of nanofiber was added to 1.5 mL of the desired buffer, as described in experimental section, at 37 ± 0.5 °C.

### 4.3. pH-Dependent Insulin Release from Nanofiber in Both Batches S1 and S2

For a typical release experiment, insulin–chitosan–poly ethylene oxide (batch S1) and S1 coated by seven-layer films on both sides of batch S2 as a sandwich contain Layer A (CS, PEG and PEO) and Layer B (PEG and PEO), as shown in Figure 1. Nanofiber was formed as result of cationic chitosan interacting electrostatically with negatively charged polymers [29] and determining how they can be used to load proteins and peptides like insulin [32]. Insulin release rates at pH values of 1.2, 6.8 and 7.4 were examined by measuring absorbance change in the supernatant at λ = 280 nm with a UV-visible spectrophotometer, as previously stated.

### 4.4. Evaluation of the Release Capability of the Insulin from Fiber

**Figure 9:**
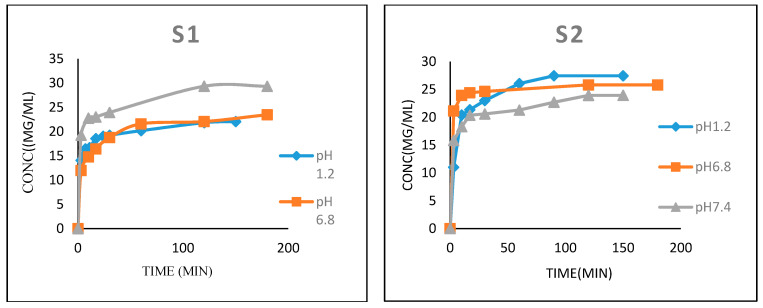
Relationship between concentration and time for batch S1 and batch S2.

The data obtained revealed that the pH has a significant impact on the release of insulin from the carrier at 37 ± 0.5 °C. Figure 9 displays three separate release experiments performed at pH values of 1.2, 6.8 and 7.4, which correspond to the stomach, colon, and blood stream habitats at body temperature, respectively. In the two batches S1 and S2, the release of insulin from nanofiber mainly involved burst release and gradual release stages. The initial fast release could be due to the encapsulated drug, which adhered to or near to the nanofiber surface that quickly detached or succumbed to pressure from the medium [33]. Donner et al. [34] reported that the half life of the pharmacokinetics of rapid insulin in the blood stream was a few minutes. The burst release takes place in about 3–5 min. The data shown in Figure 9 also show a stable plateau for batch S1 and batch S2 of insulin delivery for the stomach, colon, and blood stream. Degradation studies indicated that PEO dissolves quickly under physiological conditions, leaving behind remodeled CS fibers, implying that electrospun chitosan nanofibers could be used as an oral insulin delivery vehicle and that the PEO-coated surface exhibited hydrophobicity and low protein adsorption [35]. The strong ionic interaction between negative charges of insulin and positive charges of CS slowed the quick release of encapsulated insulin from the nanofiber at pH 1.2. Then, because of the weak affinities between the ionic groups of CS and insulin, a large amount of insulin was released at pH 6.8 and 7.4. The slight increase in insulin release detected in batch S2 could be a reminder of the presence of S1 in a sandwich between seven layers of A and B. This increases the area of the surface of PEO and PEG that may be masking and increases the distance between the positive charge of chitosan and the negative charge of insulin. Hence, there is an increase in the release of insulin. The initial release could be of immense medical advantage in the treatment or management of disease, as it constitutes the loading dose of the drug. This can result in the formation of a multidimensional entanglement network around the encapsulated drug, allowing the drug to slowly diffuse out of the release medium [33].

### 4.5. Mathematical Modeling of Release Profiles

In order to investigate the in vitro release data of insulin from nanofibers for both batches S1 and S2 at different pHs at 37 ± 0.5 °C, different kinetic models were applied. Six mathematical models, namely zero-order, pseudo-first-order, pseudo-second-order, Huguchi, Elovich, and Ritger–Peppas and Korsmeyer–Peppas equations, were used to determine the drug release kinetics for the fiber.

Comparison of the computed values for the zero-, first-, and second-order release rate constants for different pH values at 37 ± 0.5 °C for (batch-S1) and (batch-S2) nanofibers are shown in Table 3 and Table 4.

Additionally, Comparison of the computed values of the Elovich, Higuchi and Ritger–Peppas and Korsmeyer–Peppas models of insulin release for (batch-S1) and (batch-S2) nanofibers are shown in Table 5 and Table 6. for different pH values at 37 ± 0.5 °C.

### 4.6. Selection of Best Model: [20,21]

The selection of the most suitable effective model for the drug release studies is a difficult task. There are some criteria for the selection of the best suitable mathematical models, which is based upon the statistical treatments. The determination of the correlation coefficient, R^2^, is the method most widely used to assess the fit of the model equation. This method was also used when the model equation parameters were the same. The best model is the one which has the highest adjusted coefficient of determination. The kinetic data obtained for release of insulin from nanofibers for both S1 and S2 showed that the best model is the pseudo-second-order equation, for both S1 and S2, as the value of R^2^ is about one, as shown in Table 7 indicating that the solid surface has heterogeneous energy and assumes that each insulin ion is released from two adsorption sites.

**Table 3:** Comparison of the computed values for the zero-, first-, and second-order release rate constants for different pH values at 37 ± 0.5 °C for batch S1 nanofibers.

Parameter		Zero-Order Kinetic Model			First-Order Kinetic Model			Second-Order Kinetic Model			
pH	Run	Q_0_ Ppm	k_0_ × 10^3^	R^2^	k_1_ × 10^3^ min^−1^	q _e_ ppmg^−1^ (calc.)	R^2^	k_2_ g/mg min	q _e_ (calc.) ppm	h ppm g^−1^ min^−1^	R^2^
1.2	1	32.077	129	0.747	33	15.27	0.924	0.0054	47.60	11.57	0.998
	2	23.522	71	0.770	19	13.59	0.912	0.0059	32.15	6.11	0.997
6.8	1	15.163	54	0.735	30	13.21	0.899	0.0063	24.30	3.66	0.998
	2	11.530	47	0.743	24	8.08	0.916	0.0065	19.53	2.45	0.998
7.4	1	21.056	702	0.881	15	11.22	0.939	0.0076	30.20	7.00	0.997
	2	11.122	69	0.738	9	12.18	0.890	0.0071	19.30	2.60	0.991

**Table 4:** Comparison of the zero-, first-, and second-order release rate constants calculated for different pH values at 37 ± 0.5 °C for batch S2 nanofibers.

Parameter		Zero-Order Kinetic Model			First-Order Kinetic Model			Second-Order Kinetic Model			
pH	Run	Q_0_ Ppm	k_0_ × 10^3^	R^2^	k_1_ × 10^3^ min^−1^	q _e_ ppmg^−1^ (calc.)	R^2^	k_2_ g/mg min	q _e_ (calc.) ppm	h ppm g^−1^ min^−1^	R^2^
1.2	1	13.443	72	0.862	25	7.69	0.982	0.008	20.2	3.59	0.997
	2	16.607	141	0.667	28	13.46	0.960	0.006	29.4	4.97	0.998
6.8	1	23.26	17	0.527	17	2.69	0.884	0.037	26.3	24.90	1.000
	2	12.046	34	0.659	14	3.99	0.757	0.030	15.2	6.99	0.998
7.4	1	17.704	61	0.722	10	8.33	0.848	0.018	23.2	9.68	0.998
	2	15.102	31	0.879	3	10.95	0.923	0.017	18.8	6.00	0.997

**Table 5:** Comparison of the computed values of the Elovich, Higuchi and Ritger–Peppas and Korsmeyer–Peppas models of insulin release for batch S1 from nanofibers for different pH values at 37 ± 0.5 °C.

pH	Run	Elovich			Higuchi		Ritger-Peppas and Korsmeyer-Peppas		
		β g/mg	α mg g^−1^ min^−1^	R^2^	K_H_ mg g^−1^ min^−1/2^	R^2^	K	N	R^2^
1.2	Run1	0.203	436.05	0.936	0.945	0.928	0.520	0.135	0.934
	Run2	0.298	310.33	0.838	1.055	0.831	0.525	0.123	0.845
6.8	Run1	0.342	55.07	0.981	0.933	0.886	0.432	0.168	0.975
	Run2	0.401	27.26	0.954	0.810	0.891	0.321	0.181	0.965
7.4	Run1	0.418	2401.5	0.906	0.945	0.928	0.591	0.100	0.929
	Run2	0.380	23.6	0.950	0.979	0.859	0.306	0.208	0.912

**Table 6:** Comparison of the computed values of the Elovich, Higuchi and Ritger–Peppas and Korsmeyer–Peppas models of insulin release for batch S2 from nanofibers for different pH values at 37 ± 0.5 °C.

pH	Run	Elovich			Higuchi		Ritger-Peppas and Korsmeyer-Peppas		
		β g/mg	α mg g^−1^ min^−1^	R^2^	K_H_ mg g^−1^ min^−1/2^	R^2^	K	N	R^2^
1.2	Run1	0.484	228	0.969	2.189	0.934	0.526	0.133	0.977
	Run2	0.219	24.8	0.946	4.598	0.826	0.347	0.248	0.881
6.8	Run1	0.951	417,686,294	0.875	0.304	0.672	0.805	0.044	0.855
	Run2	0.966	18,633	0.791	0.434	0.761	0.648	0.079	0.790
7.4	Run1	0.641	29.56	0.944	0.772	0.853	0.044	0.100	0.947
	Run2	0.933	206.7	0.933	0.421	0.942	0.050	0.065	0.943

**Table 7:** Percentage and rate release of insulin from nanofiber in vitro. From batch S1 and batch S2 at different pH values.

	Batch S1			Batch S2	
pH	Run	% Release	K_2_ × 10^−3^	% Release	K_2_ × 10^−3^
1.2	1	95.9	5.4	90. 5	8.0
	2	96.3	5.9	90.45	6.0
6.8	1	90.0	6.3	94.26	37.0
	2	89.7	6.5	90.7	46.0
7.4	1	82.3	7.6	93.4	18.0
	2	80.2	7.1	93.4	17.0

From our studies, it can be seen that the best model is the pseudo-second-order equation, and then the rate constants were calculated from this model, as shown in Table 7. These data indicate that the rate of insulin release was stunted at pH 1.2 for both the S1 and S2 batches because of the strong ionic interaction between insulin’s negative charges and CS’s positive charges. However, there is a significant change in the rate at pH 6.8 and 7.4 due to the weak affinities between ionic groups of CS and insulin. In addition, the percentage release in batch S2 is higher, especially at pH 6.8 and pH 7.4, indicating that an improvement occurred because of the monolayer being coated with seven layers as a sandwich. By comparing the results of this study to those of previous research articles focusing on using oral insulin to protect insulin from chemical and enzymatic degradation in the stomach and small intestine, it was clear that a comparatively low amount of insulin was released from nanofibers before reaching the absorption site [36], thereby reducing the portion of insulin administrated orally that reached the blood stream to ensure control over elevated blood glucose.

## 5. Conclusions

This study provides the preparation of a multidimensional entanglement network of chitosan nanofibers around the encapsulated insulin drug, which is a major challenge for the oral delivery of insulin instead of via injection. The drug insulin released from DDS was targeted to be most probable in the colon and blood stream (simulated by an aqueous solution of pH 6.8 and 7.4) for both S1 and S2 batches since the release data were found to be strongly pH-dependent. Also, the percentages released in batch S2 were higher specially at pH 6.8 and 7.4, indicating that there is an improvement occurring due to the coating of the monolayer by seven layers. The mathematical treatment of the release data of insulin to the common kinetic models showed that the release mechanism of insulin from the prepared nanocomposite followed the rate equation of the pseudo-second order kinetic, which indicates that the solid surface has heterogeneous energy and assumes that each insulin ion is released from two adsorption sites.

## Figures and Tables

**Table 1:** Concentrations of CS, PEO, PEG, and Insulin in batch S1 and batch S2.

Layers	Cs (mg/mL)	PEO (mg/mL)	PEG (mg/mL)	In (mg/mL)
S1 “mainlayer”	16	8	-	10
A	16	8	30	-
B	-	10	25	-

**Table 2:** Mechanical properties and swelling behavior for batch S1 and batch S2 nanofibers.

Sample	Stress at Yield (Mpa)	Tensile Strength (Mpa)	Strain at Break (%)	Swelling Behavior (%)
S1	3.15 ± 0.2	4.98 ± 0.22	30 ± 3	91 ± 5
S2	6.11 ± 0.11	8.32 ± 0.30	66 ± 2	65 ± 3

## Data Availability

Data are contained within the article.
